# Coronavirus (COVID-19): A Systematic Review and Meta-analysis to Evaluate the Significance of Demographics and Comorbidities

**DOI:** 10.21203/rs.3.rs-144684/v1

**Published:** 2021-01-18

**Authors:** Arinjita Bhattacharyya, Anand Seth, Niharika Srivastava, Michael Imeokparia, Shesh Rai

**Affiliations:** University of Louisville; SK Patent Associates, LLC; University of South Carolina; SK Patent Associates, LLC; University of Louisville

**Keywords:** COVID-19, Coronavirus, SARS-CoV-2, pandemic, vaccines, clinical trials, Comorbidities, Demographics, systematic review, meta-analysis

## Abstract

**Background:**

The unprecedented outbreak of a contagious respiratory disease caused by a novel coronavirus has led to a pandemic since December 2019, claiming millions of lives. The study systematically reviews and summarizes COVID-19’s impact based on symptoms, demographics, comorbidities, and demonstrates the association of demographics in cases and mortality in the United States.

**Methods:**

PubMed and Google Scholar were searched from December 2019- August 2020, and articles restricted to the English language were collected following PRISMA guidelines. US CDC data was used for establishing statistical significance of age, sex, and race.

**Results:**

- Among 3745 patients in China, mean age is 50.63 (95% CI: 36.84, 64.42) years, and 55.7 % (95% CI: 52.2, 59.2) were males. Symptoms included fever 86.5% (82.7, 90.0), fatigue 41.9% (32.7, 51.4), dyspnea 29.0% (21.2, 37.5), cough 66.0% (61.3, 70.6), mucus 66% (61.3, 70.6), lymphopenia 18.9% (5.2, 38.0). Prevalent comorbidities were hypertension 16.4% (12.5, 20.8), diabetes 8.9% (7.0, 11.1), CVD 10.9% (6.1, 16.7), ARDS 14.6% (4.9, 27.8), malignancy 1.5 (0.05, 2.8), 1.3% (0.08, 1.9), COPD 1.3 (0.08, 1.9). 63.5 % (33.5, 88.7) received oxygen therapy, 20.8% (8.9, 35.7) were in ventilation, 23.5% (5.9, 47.8) were at the ICU. 86.5% (76.8, 94) had antiviral, 73.9% (55.3, 89.0) had antibiotics, 30% (20.6, 40.2) corticosteroids treatment.
- In the US, the odds ratio of infection in males to females is 0.873 (CI: 0.052,14.791), while the odds of dying from infection is 1.378 (CI: 0.081, 23.528) for males. The prevalence of infection is higher in females; case and death rates are higher in whites and Hispanics than other races; the death rate is higher in males irrespective of race and age; death rate per 100,000 population increases monotonically with age.

**Conclusion:**

Results showed that metabolic diseases comprising CVD, diabetes, hypertension, and respiratory diseases, including COPD, ARDS, are the most common comorbidities to severe condition and poor prognosis in covid-19 patients. Following the recent FDA’s guidance for designing Covid-19 vaccine trials, stratification factors of age, race, sex, and comorbidities need consideration in allocation. This study aimed to provide clinical researchers, health policy planners a detailed insight into the coronavirus disease.

## Introduction

In late December 2019, a series of pneumonia cases of unknown origin emerged in Wuhan, China, with clinical presentations resembling viral pneumonia. Deep sequencing analysis of lower respiratory tract samples indicated a novel coronavirus named 2019 novel coronavirus (2019-nCoV), thus creating a worldwide concern [1]. It has been more than eight months since this ‘severe acute respiratory syndrome coronavirus 2’ (SARS-CoV-2) virus has been declared a global pandemic. As we write this review, COVID-19, officially named by World Health Organization (WHO), has a case count of 21,010,700, with 761,260 deaths (as of August 14, 2020), according to John Hopkins’s Coronavirus Resource Center [2]. The United States has the highest number of cases of 5,280,315 and a death count of 167,828 as of August 2020, followed by Brazil, India, Russia. The COVID −19 pandemic is the most significant global crisis since World War-II that affected almost all the countries on Earth, according to the United Nations [3]. Unfortunately, there is a dearth of literature review on multiple topics incorporating comorbidities or subpopulation. Most of the systematic reviews focus and elaborate on a single subject area. There has been a large number of articles since the start of the pandemic; a total of 11,452 publications existed on Covid-19 from November 2019- May 2020, and it is required to identify and segregate the articles [4] for better interpretation, reproducible analysis, and understanding the bias in the research.

Here we performed a systematic review of the published literature to identify the impactful articles and review articles segregated according to comorbidities, helping clinical researchers and health policymakers allocate health resources optimally. Health care providers and health administrators can get a detailed overview of the ongoing current research relating to COVID-19 and the progress in pharmaceutical product development. Our efforts consist of summarizing the results of a broad collection of high-impact research articles that can serve as an excellent resource for current and future public health crises.

## Materials And Methods

This systematic review was conducted following the PRISMA guidelines (http://www.prisma-statement.org/). We targeted to provide a broader perspective of COVID-19 and its impact on human life, thus included several comorbidities.

### Search strategy and eligibility criteria

A systematic search of articles was done by the first author AB across PubMed/ Medline, including PubMed’s COVID-19 literature collection named

LitCovid (https://www.ncbi.nlm.nih.gov/research/coronavirus/) [5], and Google Scholar database from December 2019 up until August 2020. The articles included in the systematic search were all published studies. The studies conducted throughout the world were considered but restricted to the English language that included studies with confirmed COVID-19 cases of either sex without age restriction; The search keys to include articles for this study across all the databases were (coronavirus OR “corona virus” OR covid19 OR “covid 19” OR sarscov2 OR 2019nCoV AND (“clinical characteristics” AND “comorbidities”). Studies that were included for the meta-analysis that had the epidemiological, clinical characteristics, treatment, and outcome; explicit description of comorbidities including hypertension, diabetes, cardiovascular disease, malignancy, the symptoms such as fever, cough, fatigue, and dyspnea; a clear description of the outcomes including the significant complications such as acute respiratory distress syndrome (ARDS), and incidence of severity, hospitalization, and mortality. The exclusion criteria were the following: the absence of clinical characteristics, treatment outcome, and clinical experience.

A total of fourteen articles were included in the meta-analysis. Eighty articles were identified for inclusion based on their title and abstract that best fit each research area considered here. AB screened citations to include the highly impactful ones, and a set of eligible articles was created accordingly. Following shortlisting, the full-text articles were read thoroughly by AB, and a final set of fourteen published journal articles were included for the review, which was based on consensus among all authors. Two researchers (AB and AS) independently evaluated the included studies’ quality and cross-checked the results. Other authors (SNR and NS) also participated in the discussion, whenever necessary, and thoroughly studied the included studies. Correspondences, letters, and editorial comments were excluded, as they were devoid of numeric data, but some have been assessed narratively. Some articles from the reference lists of other articles were also identified. The statistical testing for heterogeneity was conducted to determine the prevalence of comorbidities, sex, symptoms in selected studies for meta-analysis. The meta-analysis of proportions with 95 % CI was calculated for the clinical symptoms, comorbidities, treatments, and outcomes using R statistical software with R software’s ‘meta’ and ‘metafor’ packages. The null hypothesis of no heterogeneity between studies was rejected with p-value <0.05. A random-effects model was applied to conduct the meta-analysis with heterogeneity statistics: τ^2^ representing between-group variation and I^2^ being a proportion of total variation visualized by forest plots. The individual confidence intervals are shown, by study, with each group’s proportion and confidence interval. A random-effects model was used for the combined proportion to check for heterogeneity with I^2^ = (25%, 50%, 75%) represented low, medium, and high heterogeneity.

## Global Results

**Fig 1:**
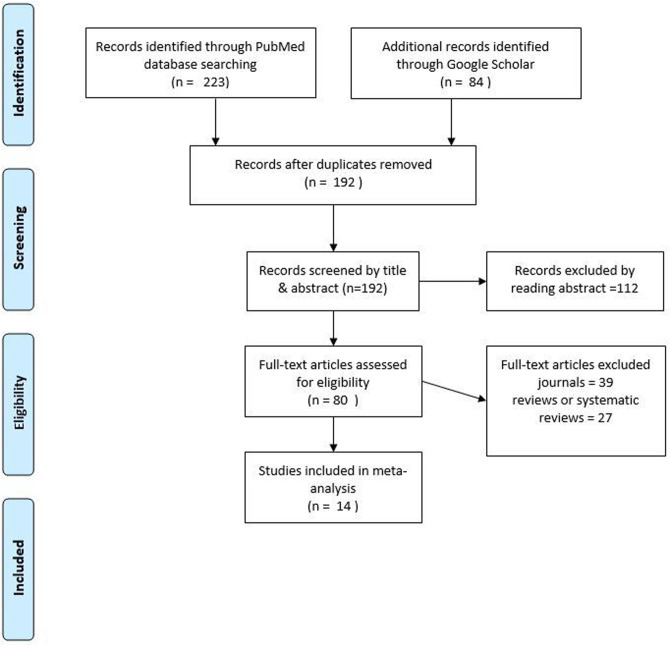
PRISMA flow diagram

The PRISMA diagram details the selection of articles in Fig 1. In total, eighty articles were evaluated for inclusion in the article, of which sixty-six are excluded as they were either systematic review articles, reviews, editorials, journal articles, perspectives, or correspondences. Some journal articles are not included in the quality analysis as they were not relevant to the primary outcome. A total of 14 articles were included for meta-analysis.

**Fig 2:**
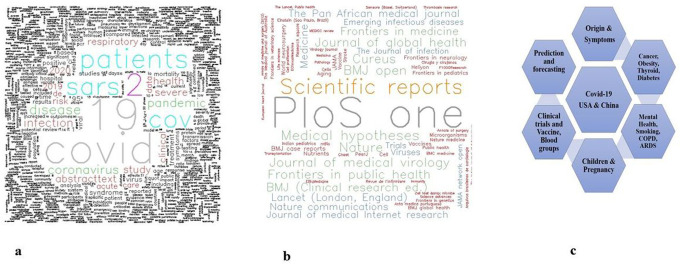
Wordclouds of (a) abstracts & (b) journals (c) Comorbidities considered in research

In Fig 2, word clouds from abstracts and journals of recently published articles help visualize their frequency.

Among 3745 patients in China, mean age is 50.63 (95% CI: 36.84, 64.42) years, and 55.7 % (95% CI: 52.2, 59.2) were males. The common symptoms included fever 86.5% (82.7, 90.0), fatigue 41.9% (32.7, 51.4), dyspnea 29.0% (21.2, 37.5), cough 66.0% (61.3, 70.6), mucus 66% (61.3, 70.6), lymphopenia 18.9% (5.2, 38.0). Prevalent comorbidities were hypertension 16.4% (12.5, 20.8), diabetes 8.9% (7.0, 11.1), CVD 10.9% (6.1, 16.7), ARDS 14.6% (4.9, 27.8), malignancy 1.5% (0.05, 2.8), COPD 1.3% (0.08, 1.9). 63.5 % (33.5, 88.7) of covid-19 patients received oxygen therapy, 20.8% (8.9, 35.7) were in ventilation, 23.5% (5.9, 47.8) were at the ICU. 86.5% (76.8, 94) patients were treated with antiviral, 73.9% (55.3, 89.0) had antibiotics, 30% (20.6, 40.2) had corticosteroids treatment. 32.3% (21.3, 44.5) were discharged, 52.6% (43.1, 62) remained in hospital, and 7.5% (2.6, 14.4) died.

The next section provides a narrative review of the inception of the pandemic, focusing on specific comorbidities.

### Origin of COVID-19

**Fig 3.**
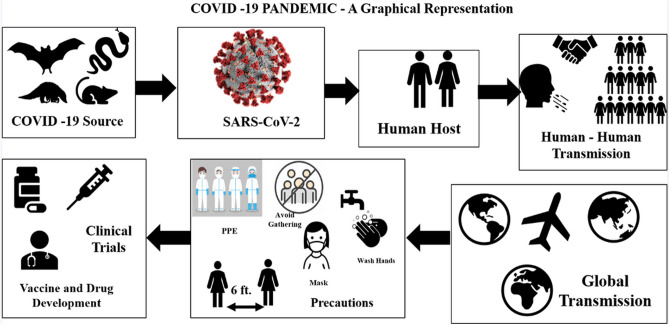
COVID-19 Pandemic - A Graphical Representation

Coronaviruses are not new to human society. It has occurred previously in China in the form of Severe Acute Respiratory Syndrome (SARS) and infected over 8000 persons and 776 deaths in 2003 [6] and was named SARS-COV [7, 8]. The Middle East Respiratory Syndrome Coronavirus (MERS-CoV) was detected in Saudi Arabia in 2012 [9], infecting more than 2428 individuals and 838 deaths across 27 countries, as reported to WHO. Initial Coronavirus 2019 (COVID-19) cases began at the Huanan wholesale seafood market in China that traded live exotic animals, and since then, it has severely affected humanity. The virus was identified by genome sequencing as a coronavirus with 96.2% homology (genetic identity) with bat coronavirus (CoV RaTG13) and 79.5% similarity with SARS-CoV. Humans’ transmission is through an unknown intermediate source, which scientists have suspected to be bat, swine, snake, civet, pangolin, or mouse, among others [10–12]. Identifying a potential intermediate host of SARS-CoV-2 from the SARSCoV-2 genome features can lead us to the proximal origin of and spread of the virus, from animal species boundaries to human. It is not a laboratory construct virus. [13]. Recent research finds SARS-CoV-2 bat sarbecovirus RaTG13 as the origin, claims of pangolin [14] being the intermediate host, or the virus being laboratory construct have been discarded [15]. It has been estimated that the virus has a higher reproductive number (R_0_) compared to SARS [16], which is around 3.28, with a median of 2.79. Fig 3 gives an overview of the pandemic explaining its origin, transmission, and the recommended precautions.

### Clinical Features and Symptoms

**Fig 4:**
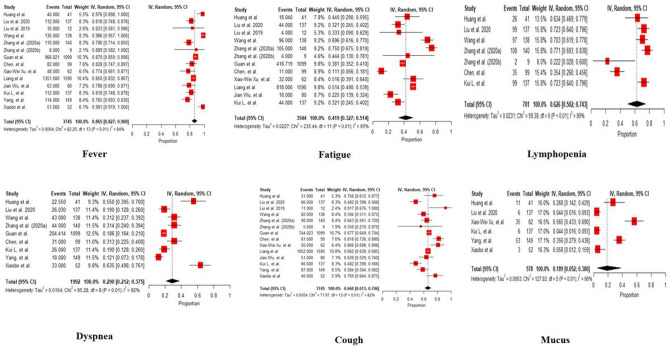
Forest plot of the proportion of common symptoms

The incubation period of the virus is 2–14 days; however, it is frequently 3–7 days [17, 18]. It is highly contagious and does get transmitted from human-to-human contact, which has led to a high number of deaths. The human-to-human spreading of the virus occurs due to proximity with an infected person or patients or an individual exposed to coughing, sneezing, and respiratory aerosols droplets. These aerosols penetrate the human body via inhalation through the mouth or nose. In the study by Guan et al., patients’ median age was 47–59 years, and 41.9–45.7% were females in China [19]. The symptoms include fever, cough, fatigue, sputum production, shortness of breath, sore throat, headache, diarrhea, and nausea or vomiting [19, 20]. Cases that involve symptoms like breathing trouble, persistent pain, or pressure in the chest, confusion, bluish lips require emergency medical attention (www.cdc.gov). People over 65 years and those with underlying disorders (i.e., hypertension, chronic obstructive pulmonary disease, diabetes, cardiovascular disease) were more prone to rapidly develop acute respiratory distress syndrome, septic shock, metabolic acidosis, and coagulation dysfunction, often resulting in death [1]. The proportion of typical symptoms are represented in Fig 4.

### COVID-19 and Comorbidities

**Fig 5:**
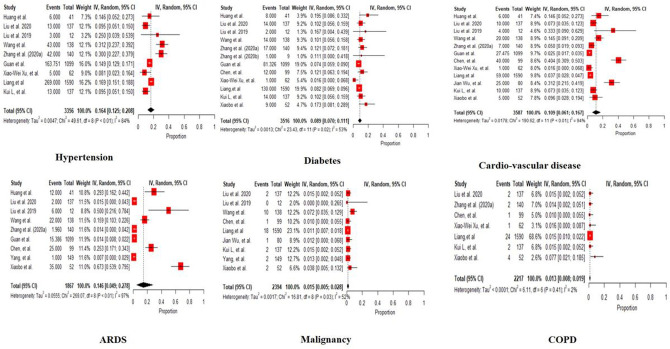
Forest plot of the proportion of Comorbidities

People with associated comorbidities and preexisting conditions, including heart failure, uncontrolled diabetes, COPD, asthma, and cancer, are the risk group who would require to be put on ventilator support or oxygen therapy due to the COVID-19 event of the hospitalization. The effects of COVID-19 and its adversity due to comorbidities are explained in Fig 5 with Table 1 comparing the proportions among three tremendously affected countries of China, India, and the US.

In all the forest plots, total patients differ for each outcome differ as there was missing data.

### Acute Respiratory Distress Syndrome

Pre-clinical and clinical studies indicate that ARDS is a primary manifestation in COVID-19 patients [21]. Among a large group of hospitalized patients who died, three-fourth of them developed ARDS, a significant of them required ICU and invasive ventilation [22].

### Diabetes

Patients with pre-diabetic conditions and severe metabolic complications are prone to an increased risk of severe outcome from COVID-19, thus requiring blood glucose control [23]. Also, COVID-19 may trigger diabetes in healthy people resulting in a bidirectional relationship between the two diseases; SARS-CoV-2 may cause pleiotropic alterations of glucose metabolism due as it binds with ACE2 expressed in essential metabolic organs, tissues, kidneys that could complicate conditions of preexisting diabetes or lead to new mechanisms of disease. [24]. Recent initiatives have been taken by an international group of leading diabetes researchers in the COVIDIAB project (covidiab.e-dendrite.com) to study the new-onset COVID-19 related diabetes, along with data collection on preexisting metabolic disturbances including diabetic ketoacidosis, hyperosmolarity. Diabetes mellitus was associated with the critical outcome of severe COVID-19 disease progression and mortality, which was also influenced by age, obesity, and hypertension [25, 26].

### Obesity

The scarcity of Body Mass Index (BMI) data on COVID-19 patients’ needs special attention in understanding the underlying relation of obesity to COVID-19 infection and its severity due to obesity-induced adipose tissue inflammation in type 2 diabetes, hypertension, cardiovascular disease[27]. Obesity can lead to decreased expiratory reserve volume, functional capacity, and respiratory system compliance. Also, previous hospitalizations due to the H1N1 virus were higher in obesity, prevalent populations of Americans Indians, Hispanics, African Americans. The adult obesity rate has increased since the time of H1N1 in 2009. Thus, indicating the severe course of COVID-19 in such groups than H1N1 [28]. This evidence draws the need for precautions, prioritizing testing, care, and expanding research in people with obesity and COVID-19 to develop an effective treatment. It is observed that most of the younger COVID-19 hospitalized population were suffering from obesity [29]. The presence of morbid obesity (BMI ≥ 35 kg/m^2^) aggravates the respiratory disease and can result in intensive care unit (ICU) admission [30].

### Cancer

It is crucial to understand the effects of COVID-19 and its associated and potentially serious outcomes, high susceptibility, and treatment effects specifically for cancer patients. More data-driven and broad collaborative efforts are underway like TERAVOLT, CCC19, ASCO Registry, among others [31]. The significant risk for cancer patients lies in the inability to get necessary treatment, medications as the healthcare workforce being concentrated towards treating patients with COVID-19, delay in oncology clinical trials due to delayed or canceled enrollment appointments with two-hundred international trials suspended between March to April [32], and restrictions on hospital visits, delayed surgeries due to the present infection [33, 34]. Thus cancer treatments have been modified to align with the situation, such as delaying breast cancer screening and lung cancer screenings to decrease the risk of exposure, the use of personal protective equipment (PPE) in surgery rooms, and implementing the RADS (Remote visits, Avoid radiation, Defer radiation, Shorten radiation) principle [35]. Cancer patients show adverse outcomes after coronavirus infection than other healthy individuals [36]. For immunosuppressed patients, personal level control measures for COVID-19, vigorous and robust screening and monitoring, and health care improvement are the recommended strategies. There should be an emphasis on the precautionary principle, evidence-based prioritizing of treatments while treating cancer patients during this pandemic [37] since they are at increased risk of COVID-19 [38, 39]. There is a need for clear communication and education about hygiene, infection, and control measures, with consideration of postponing surgery in patient groups with the standard progression of cancer, recommending the use of telemedicine [38].

### Thyroid

There is not enough significant data that suggests that patients with preexisting thyroid conditions are more susceptible to COVID-19, and there is no general guidance, unlike other comorbidities. There have been instances of thyroid dysfunction during SARS, and risks of anti-thyroid drugs resulting in agranulocytosis remain, as the symptoms resulting from agranulocytosis and COVID-19 are similar, making it difficult for doctors to distinguish between the two [40]. A study in China found that the decrease in TSH and TT3 levels positively correlated with the severity of the COVID-19 disease, with lower TSH levels present in more than half of the patients. This change in serum TSH and TT3 levels will help to diagnose the course of COVID-19 [41]. There is evidence of some COVID-19 patients suffering from ear pain, which may indicate a sign of subacute thyroiditis along with tachyarrhythmia as the most common cardiovascular disease, so monitoring of thyroid function in COVID-19 patients is essential [42]. Patients with thyroid eye disease and those undergoing treatment with immunosuppressive agents are at a very high risk of severe illness from coronavirus. Pregnant women with treated hypothyroidism should continue to follow the advice to increase levothyroxine dose and regularly check their thyroid levels [43].

### Smoking

COVID-19 is mainly a disease of the respiratory tract characterized by severe acute respiratory syndrome. Studies showed that smokers are 1.4 times likely to have severe symptoms of COVID-19 and approximately 2.4 times more likely to be admitted to an ICU, need mechanical ventilation, or die compared to non-smokers [44].

### Chronic Obstructive Pulmonary Disease

Preexisting chronic obstructive pulmonary disease (COPD) and ongoing smoking history are likely to worsen the progression of symptoms and treatment effectiveness for COVID-19 [45]. Compared to previous and non-smokers, current smokers are more susceptible to severe complications and higher mortality rates, requiring preventive measures [46].

### Mental health

Research-based evidence suggests that anxiety and depression symptoms and self-reported stress (8%) are common psychological reactions to the COVID-19 pandemic, as there is limited interaction with the outside world. Due to the lockdown, social distancing people, financial loss, confinement within homes, mental health is likely to get impacted. There has been an increased domestic abuse rate and family violence reporting due to confinement by social isolation and quarantine measures [47]. It is very likely that the healthcare workers may experience mental health problems due to their work under nerve-wracking pressure, and need to make decisions under unprecedented conditions of scant resources, life-support and intensive care distribution to the ones those have a higher scope of survival over equally needy elderly patients[48]. Large and growing financial loss, conflicting messages and guidelines from authorities, travel and visa restrictions may be a possible cause of mental distress and anxiety and calls for immediate action[49, 50]. It is suggested that along with health care providers, children, residents of remote rural areas, immigrants, especially international student populations, should be explored. Few studies have suggested psychologists and psychiatrists’ critical role in healing people’s mental health affected by this pandemic and those who survive COVID-19 [51].

### Blood Type and COVID-19

Several studies have identified that blood group is a risk factor for COVID-19 infection. People with blood groups A, B, and AB have an increased risk of infection, while people with blood group O have a decreased risk of infection, possibly due to less blood clotting. [52]. Table 2 provide the distribution of the blood group in the reference population and Covid-19 risk associated with the blood group in a few selected studies

It is interesting to note that the odds ratios for blood groups A, B, AB, and O in the three Chinese studies are similar. Based on these three studies, there is a 32%−35% chance reduction in the O group for Covid-19 patients. In the other two studies, the risk of getting Covid-19 infection in patients with blood group O is less than the other blood groups.

### Pediatric Group and COVID-119

Children have represented 2% of diagnosed cases in China, 1.2% in Italy, and 5% of positive cases in the United States that increased to 12% as of December 19, 2020[57]. The proportion of infection among children may be milder compared to adults with better prognosis response and rare death rates [57]; however, SARS COVID-19 infections among children were occurring early in the pandemic [58], and better quality studies are required for judging increased risk for severe inflammation and multiorgan failure. Studies suggest that children of all ages are susceptible to the infection, with a significant role in community-based virus transmission [59], and the number of pediatric patients may increase in the future unless stringent measures are undertaken [60]. A recent meta-analysis based on 266 relevant articles and letters showed that children have milder than adults symptoms but with variable manifestations and significantly better mortality outcome [61].

### Pregnancy during COVID-19

Based on a small group of cases, currently, there is minimal evidence for intrauterine infection caused by vertical transmission or transplacental transmission in women who develop COVID-19 pneumonia during late pregnancy with varied symptoms, including cough and fever [62]. A single case of severe maternal presentation makes it impossible to rule out the possibility of vertical transmission [63, 64]. Currently, there is no evidence to believe that pregnant women are at increased risk [65], but this group should be considered as a vulnerable population and may have more severe respiratory complications [66].

Immune, respiratory, and cardiovascular systems are affected in pregnancy due to physiological changes. COVID-19 infection during pregnancy may adversely affect these systems. A recent update on the direct and indirect effect of COVID-19 on pregnancy is provided by Wastnedge et al. 2021 [67].

### Vaccines and Therapeutics development for COVID-19

Multiple pharmaceutical companies are working for the development of effective COVID-19 vaccines, such as Moderna Therapeutics, Inovio Pharmaceuticals, Novavax, Vir Biotechnology, Stermirna Therapeutics, Johnson & Johnson, Pfizer, Merck, Glaxo-smith Kline, VIDO-InterVac, GeoVax-BravoVax, Clover Biopharmaceuticals, CureVac, and Codagenix to mitigate the spread of this infectious disease.

Research institutes all over the world are working relentlessly to find a cure for COVID-19. According to the National Institute of Allergy and Infectious Diseases (NIAID) director Dr. Anthony S. Fauci, the idea of vaccine emerging by the end of the year is “aspirational, but it is certainly doable.” A vaccine or any drug has to go through the four phases of clinical trials before it is approved by the US Food and Drug Administration’s (FDA’s) or similar authorities for human use, while continuously balancing the safety, efficacy, and side effects due to the vaccine exposure.” The most significant risk concern is the transmission of infection to healthcare workers. The first case in the United States occurred on January 19. The patient’s condition improved by remdesvir, an investigational broad-spectrum antiviral treatment developed by Gilead Sciences, Inc. [68]. A randomized, controlled clinical trial evaluating the safety and efficacy of treatment with antiviral remdesivir for coronavirus disease 2019 (COVID-19) was initiated by (NIAID) on February 15 via Adaptive COVID-19 Treatment Trial (ACTT) [69]. It has been statistically significant in shortening the recovery period for those who have received remdesivir rather than the placebo (median, 11 days, compared with 15 days) [70]. The next trial, ACTT2 with remdesivir with Baricitinib, a product licensed to Eli Lilly company by Incyte, will determine if this addition can benefit patient recovery [71]. Chloroquine has been used to treat malaria over the years. Chloroquine and hydroxychloroquine have a wide range of potential in treating SARS CoV [72]. A clinical trial is ongoing to establish its utilization in the treatment [73]. It was recommended to be included as a treatment due to its apparent efficacy and acceptable safety against COVID-19 associated pneumonia, improving lung image findings, shortening the recovery period, and blocking the infection in in-vitro studies according to multicenter clinical trials in China [74]. Finally, researchers from Brazil established no distinguished improvement due to the drug after 15 days from infection in a large-scale clinical trial consisting of 504 COVID-19 patients. They concluded that the safety and efficacy of using hydroxychloroquine in treating COVID-19 patients are limited [75]. Protease inhibitors lopinavir and ritonavir used to treat the infection with human immunodeficiency virus (HIV) [76] has been recommended by researchers to help in the recovery of MERS-CoV [77] and SARS-CoV [78]. Studies claim that after lopinavir/ritonavir (KALETRA^®^, AbbVie) was administered, β-coronavirus viral loads significantly decreased, and no or little coronavirus titers were observed in treating the index patient in Korea [79]. Recent clinical trials to identify the efficacy and safety of oral Lopinavir–Ritonavir for COVID-19 did not significantly benefit recovery, reducing mortality, or diminishing throat viral RNA in critically ill COVID-19 patients[80]. Others include traditional Chinese medicine, combined Chinese medicine Shufeng Jiedu capsule (SFJDC), and western medicine [81]. The use of traditional Chinese medicine in H1N1 influenza and SARS prevention based on historical records leads it to be a prospective alternative for preventing COVID-19 in a high-risk population. The most frequently used herbs included Radix astragali (Huangqi), Radix glycyrrhiza (Gancao), Radix saposhnikoviae (Fangfeng), Rhizoma Atractylodis Macrocephalae (Baizhu), Lonicerae Japonicae Flos (Jinyinhua), and Fructus forsythia (Lianqiao) [82, 83]. The chimpanzee adenovirus-vectored vaccine (ChAdOx1 nCoV-19) has extremely promising results with an acceptable safety profile and homologous boosting increased antibody responses developed by the Oxford- AstraZeneca vaccine group [84]. Given the race of finding a treatment for COVID-19 and the accelerated rate of clinical trials, a real-time dashboard is available to track the clinical trials and understand their importance [85]. Ongoing clinical trials for the prevention and successful intervention are recorded [86, 87]. Vaccine development is a lengthy process, and though unlikely, in the event of an abrupt ending of the pandemic, promising vaccine candidates should be developed as preparation for another possible outbreak [88].

### Prediction Models and COVID-19

There has been an ocean of research articles on prediction and models supporting medical decision making, diagnostic and prognostic efforts. Prediction modelling efforts include standard epidemiological models like Susceptible-Infectious-Recovered-Dead (SIDR) [89], Susceptible-Exposed-Infectious-Removed (SEIR) [90], extended state-space SIR (eSIR) [91], artificial intelligence, machine learning [92], and deep learning [93]. A review of the current literature pool to assess the diagnostic and prognostic multivariable prediction models of COVID-19 found common predictors like age, temperature, signs and symptoms, blood pressure, sex, comorbidities, and creatinine. It also reflects that all models included in the study reported optimistic performance, with a high risk of bias with poor reporting and predictor description, thus requiring rigorous prediction models supported by methodology. Cautious use of these models in current practice is recommended [94].

Twenty-seven review articles were separated based on origin, symptoms, comorbidities like ARDS, COPD, mental health, diabetes, obesity, cancer, smoking, pregnancy, children, thyroid, vaccine, therapeutics, and prediction models. Additional file 1 provides the review category, primary outcomes, no. of included studies, sample size, and conclusion for all the 27 main review articles. Methodological quality assessment of the included literature reviews was performed using the AMSTAR tool [95]. The AMSTAR score consists of a total of 11 points. A study is considered high quality with a score (9–11), medium quality with a score (6– 8), and low quality with a score (0–5).

## Usa Results

### Significance of Demographic Variables: Sex, Race, and Age

Based on data from the CDC website concerning the demographics (from inception – August 10, 2020) of deaths and cases in the US, we have tested the hypothesis of the significance of gender, race, and age and their impact on the death and case rates by the binomial proportion test. Demographics characteristics for the US population are statistically significant. In Table 3 concerning the gender difference, the infection rate is higher in females, while the death percentage is more significant in males than females.

The odds ratio of the case rate in males concerning females is 0.873 (CI: 0.052,14.791), while the death rate is 1.378 (CI: 0.081, 23.528). The risk ratio of males vs. females case rate is 0.934 and 1.173 for the death rate. The death rate per 100,000 population is presented in Table 4a by race, and their ranking is available.

The cases and death per 100 000 are highest in White Non- Hispanic (203.2; 15.19) group, followed by Hispanic/ Latino (164.92;5.17) and Black Non- Hispanic (104.36;6.81) groups.

The differences between pairs of race groups are tested for statistical significance, and all the two-pair tests have resulted in p-values <0.05, indicating race is a critical factor in Table 4b.

**Fig 6:**
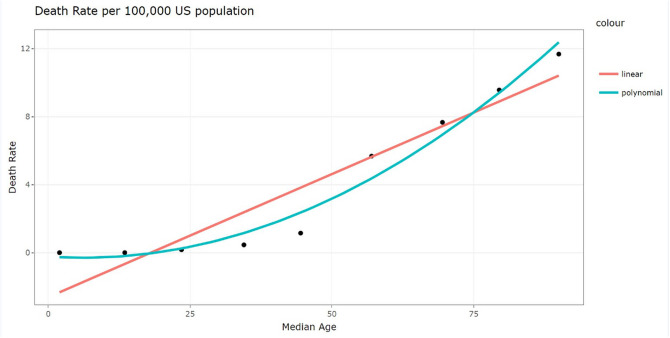
Fitted Growth Curve to the Death Rate per 100,000 population

In Table 5a regarding age, a monotonic increase in deaths per 100,000 population with the increase in age is apparent shown by fitting linear and polynomial curves to the death rates, illustrated by Fig 6.

Table 5b shows a definite distinguished statistical significance of cases and deaths among three age groups. Such a clear demonstration of mortality rates by age should undoubtedly help the health policy planners in designing appropriate health/insurance plans.

The p-values in all the following Table 3–5 are much smaller (10^−8^) than 0.05 as the sample sizes in each of the groups are large, denoting that all three factors (race, gender, and age) are significant.

## Discussion

To the best of our knowledge, this is the first research article in the COVID-19 literature to expand over such a wide range of subject areas. All the articles reviewed are of high quality, leaving a few with non-availability of data or detailed narratively.

COVID-19 has rapidly spread since its initial identification in Wuhan. Literature and information on this newly emerged coronavirus disease are published at a pandemic speed, so an attempt has been made to cluster articles on a similarity basis [96]. Evidence maps have been created to identify the benefits of medical literature [97]. Among the preexisting health conditions, cardiovascular disease, hypertension, metabolic disease, and obesity among adults are the primary conditions among cases as of August 2020.

Recent developments have been highly promising with the approval of the vaccine’s emergency use from Pfizer-BioNTech [98] among health care workers, first responders. In five months from August-Dec and the increasing number of cases, deaths, the US entering the second wave of the pandemic, there has been immense progress with potential vaccine candidates of Moderna, AstraZeneca therapeutics development. Vaccines for children (under 18), pregnant women, their distribution in the world, dealing with the new variant of the virus found in the UK are few among many ongoing concerns to overcome relating to the pandemic.

## Conclusion

Early quarantining, early diagnosis and immediate treatment might collectively contribute to a better outcome for the patient. Mass testing [99], complete isolation of infected patients, and the use of face masks [100] are crucial to interrupt the spread of the virus, flatten the curve, prevent the drastic economic decline, unprecedented public health challenges, and the risk of second wave infection [101]. The effect and impact of COVID-19 are heterogeneous and vast and difficult to be confined within a single literature review; however, our review will give a broad and generic overview to the researchers and the public. Clinical trial researchers may look into statistical design and recommendations for Phase II/III clinical trials to test therapeutic interventions of COVID-19 [102]. The COVID-19 has been the greatest threat to people’s health and safety due to its high contagious nature. The virus has unfortunately spread to more than 213 countries, leading to national emergencies, shut down of airports, travel ban, and complete lockdown in countries like Italy, Germany, China, Canada, India, among others.

Precautions and educating the masses about social distancing and protective gear should be undertaken with utmost urgency to repeat the unfortunate and gravest incident of the 1918 pandemic. Our systematic review on COVID-19-positive patients will help healthcare researchers, providers, health policy executives and, health policy/insurance planners to clearly understand the broader picture of the pandemic and implement updated treatment strategies and mitigate the COVID-19 pandemic and its health, psychological, economic, and medical consequences. Our research finds that race, age, sex, and comorbidities have influenced the outcome of covid-19 patients. It is recommended that these factors are considered in designing clinical trials, as also reflected in the recently published FDA guidance for diversity inclusion in conducting clinical trials [103].

### Limitations

This review has few limitations to note. The tremendous volume of evolving research makes it challenging to review multiple outcomes on a large scale. Second, in a few areas like thyroid, obesity, very few articles were available. Third, the articles were limited to English-language publications., the other high impactful or influential studies relevant but published in other languages could be lacking. Most of the studies included in our review are from China and the United States. Fourth, there are some overlap within studies due to the wide variety of outcomes considered and inter-relation between comorbidities such as (COPD and smoking history), (thyroid, obesity, diabetes), (diabetes and ARDS).

## Supplementary Material

Supplement

## Figures and Tables

**Table 1:** Comparison of the proportion of comorbidities globally

Comorbidities	China (N)	China % (CI)	US (%)*	India (%)**
Hypertension	3356	16.4 (12.5,20.8)	21.6	42.5
Diabetes	3516	8.9 (7.0,11.1)	16.4	39.7
CVD	3587	10.9 (6.1,16.7)	34.3	NA
ARDS	1867	14.6 (4.9,27.8)	12.9	NA
Malignancy	2394	1.5 (0.5,2.8)	4.6	NA
COPD	2217	1.3 (0.8,1.9)	23.59	16.4

* US data from CDC website till October 24, 2020;

** 522 patients [21]

**Table 2:** Distribution of Blood type in literature

Blood Group	Zhao et al. 2020[53] (China)		Wu et al. 2020 [54] (China)		Li et al. 2020 [55] (China)		Goker et al. 2020 [56] (Turkey)		Barnkob et al. 2020 [52] (Denmark)	
	%	OR	%	OR	%	OR	%	OR	%	RR
A	32.2	1.28	27.4	1.54	32.2	1.30	38.0	2.1	42.4	1.09
B	24.9	1.09	NA	NA	24.9	1.06	14.7	1.4	11.4	1.06
AB	9.1	1.11	NA	NA	9.1	1.13	10.0	1.3	4.2	1.15
O	33.8	0.68	30.2	0.65	33.8	0.68	37.2	1.8	41.7	0.87

OR=Odds Ratio, RR=Relative Risk, NA= Not available

**Table 3:** COVID-19 Cases & Deaths by Sex Significance

Sex	Cases^a^		Deaths^b^	
	%	p-value	%	p-value
Female	51.7	< 10^−8^	46	< 10^−8^
Male	48.3		54	
Other	<0.1		<0.1	

a Data from 3,855,323 cases. Sex was available for 3,756,128 (97%) cases

b Data from 124,414 deaths. Sex was available for 123,567 (99%) deaths

**Table 4a:** COVID-19 Cases & Deaths by Race

Race/Ethnicity	Case %	Case Counts	Cases per 100,000	Rank by Case %	Death %	Death Counts	Deaths per 100,000	Rank by Death%
White Non-Hispanic	39	672603	203.2	1	50.0	50270	15.1	1
Hispanic/Latino	31.6	545892	164.9	2	17.0	17110	5.1	3
Black Non-Hispanic	20	345424	104.3	3	22.4	22531	6.8	2
Multiple/Other Non-Hispanic	4.2	71851	21.7	4	4.6	4628	1.4.0	5
Asian Non-Hispanic	3.7	63694	19.2	5	5.1	5127	1.5	4
American Indian / Alaska Native Non-Hispanic	1.3	21929	6.6	6	0.7	745	0.2	6
Native Hawaiian / Other Pacific Islander Non-Hispanic	0.3	5539	203.2	7	0.1	140	0.0	7

Data from 3,620,197 cases. Race/Ethnicity was available for 1,726,932 (48%) cases

Data from 120,593 deaths. Race/Ethnicity was available for 100,551 (83%) deaths

US Population: 331,002,651

**Table 4b:** Difference between Races with COVID-19 Cases and Deaths

Group1	Group2	P-values for Cases	P-values for Deaths
Comparison with the White (Non-Hispanic) Group			
White	Hispanic	< 10^−8^	< 10^−8^
White	Black	< 10^−8^	< 10^−8^
White	Multiple	< 10^−8^	< 10^−8^
White	Asian	< 10^−8^	< 10^−8^
White	American Indian	< 10^−8^	< 10^−8^
White	Native Hawaiian	< 10^−8^	< 10^−8^
Comparison with the Black (Non-Hispanic) Group			
Black	Hispanic	< 10^−8^	< 10^−8^
Black	Multiple	< 10^−8^	< 10^−8^
Black	Asian	< 10^−8^	< 10^−8^
Black	American Indian	< 10^−8^	< 10^−8^
Black	Native Hawaiian	< 10^−8^	< 10^−8^
Comparison with the Hispanic Group			
Hispanic	Multiple	< 10^−8^	< 10^−8^
Hispanic	Asian	< 10^−8^	< 10^−8^
Hispanic	American Indian	< 10^−8^	< 10^−8^
Hispanic	Native Hawaiian	< 10^−8^	< 10^−8^
Comparison between other Groups			
Asian	American Indian	< 10^−8^	< 10^−8^
American Indian	Native Hawaiian	< 10^−8^	< 10^−8^

**Table 5a:** COVID-19 Cases & Deaths by Age

Age Group	Case Percentage	Case Counts	Cases per 100,000	Death Percentage	Death Counts	Deaths per 100,000
0 – 4 Years	1.6	55717	16.8	<0.1	29	0.0
5–17 Years	5.8	201486	60.8	<0.1	48	0.0
18–29 Years	21.6	751715	227.1	0.5	603	0.21
30–39 Years	17.1	596121	180.1	1.3	1554	0.5
40–49 Years	15.8	551015	166.4	3.2	3833	1.2
50–64 Years	21.7	755186	228.15	15.6	18814	5.68
65–74 Years	8	277847	83.94	21.1	25377	7.67
75–84 Years	4.7	163214	49.31	26.3	31677	9.57
85+ Years	3.7	127556	38.54	32.1	38645	11.68

Data from 3,620,197 cases. Age group was available for 3,479,857 (96%) cases;

Data from 120,593 deaths. Age group was available for 120,580 (99%) deaths

**Table 5b:** Difference between Age Groups with COVID-19 Cases and Deaths

Age Groups	P-values for Cases	P-values for Deaths
<= 17 Yrs. & 18–64 Yrs.	< 10^−8^	< 10^−8^
18–64 Yrs. & >= 65 Yrs.	< 10^−8^	< 10^−8^
>= 65 Yrs. & <= 17 Yrs.	< 10^−8^	< 10^−8^
