# Supplementary material for: Coronavirus (COVID-19): A Systematic Review and Meta-analysis to Evaluate the Significance of Demographics and Comorbidities
Source: Res Sq. 2021 Jan 18:rs.3.rs-144684. Preprint. [Version 1] doi: 10.21203/rs.3.rs-144684/v1 (PMC7814834; doi:10.21203/rs.3.rs-144684/v1)
Supplement: Supplement [file 32bf8ccaf01c2b79602f0889.docx]

Table S1: Characteristics of Reviews Included for Quality Analysis

| **Reference** | **Topic** | | | **Date Range** | | **Review Category** | | **No. of included studies** | | **Sample Size** |
| --- | --- | --- | --- | --- | --- | --- | --- | --- | --- | --- |
| [7] | Origin, transmission, and characteristics of human coronaviruses | | | July, 2020 | | review | | 14 | | NA |
| - Primary Outcome: Comparison of analysis of the emergence and pathogenicity of COVID-19 infection and previous human coronaviruses - Main Conclusion: Hunan seafood market at Wuhan, China; zoonotic source not confirmed; sequence-based analysis suggested bats as the key reservoir. | | | | | | | | | | |
| [11] | origin, transmission, and clinical therapies | | | Inception -March 1,2020 | | review | | NA | | NA |
| - Primary outcome: origin, transmission, clinical characteristics, and clinical therapies - Main Conclusion: Originating from the reservoir of bats and unknown intermediate hosts, SARS-CoV-2 binds to ACE2 with high affinity as a virus receptor to infect humans | | | | | | | | | | |
| [14] | Proximal origin | | | Inception -11 March 2020 | | review | | 5 | | NA |
| - Primary outcome: the origin of SARS-CoV-2 from a comparative analysis of genomic data - Main Conclusion: SARS-CoV-2 is not a laboratory construct or a purposefully manipulated virus | | | | | | | | | | |
| [17] | reproductive number (R0) | | | 1 Jan 2020 – 7 Feb, 2020 | | review | | 12 | | NA |
| - Primary outcome: reproduction number (R0) of the COVID-19 virus - Main Conclusion: The reproductive number of COVID-19 is higher compared to SARS | | | | | | | | | | |
| **Symptoms of COVID-19** | | | | | | | | | | |
| [23] | Clinical, laboratory and imaging features | | | January 1 2020 – February 23, 2020 | | Systematic review  & meta-analysis | | 19 studies, 39 case reports | | 656 |
| - Primary Outcome: Fever, cough, and dyspnea as the most critical manifestations - Main Conclusion: Patients with chronic medical conditions are a high-risk group with important symptoms, including rapid progression to ARDS. | | | | | | | | | | |
| [91] | Imaging Findings | | | Until March 14, 2020 | | Systematic review  & meta-analysis | | 30 | | 919 |
| - Primary outcome: initial CT includes bilateral multilobar ground-glass opacification (GGO) with a peripheral or posterior distribution, mainly in the lower lobes and less frequently within the right middle lobe. - Main Conclusion: Provides initial and follow-up CT characteristics of the disease | | | | | | | | | | |
| **COVID-19 and Pediatric group** | | | | | | | | | | |
| [55] | Effect of COVID-19 on children | | | Jan 1-March 18, 2020 | | systematic review & meta-analysis | | 45 case studies | | 1%-5% of diagnosed |
| - Primary Outcome: Symptoms and prognosis in children - Main conclusion: children show milder cases and a better prognosis than adults, deaths are sporadic | | | | | | | | | | |
| [92] | School closure and management | | | March 19, 2020 | | Rapid systematic review | | 16 articles | | NA |
| - Primary Outcome: Effect of school closures - Main conclusion: school closures for COVID-19 and combinations of social distancing measures should be considered to mitigate death rate | | | | | | | | | | |
| **COVID-19 on Pregnancy** | | | | | | | | | | |
| [93] | | Pregnant patients with Covid-19 | March 14 – March 16, 2020 | | Systematic review | | 6 retrospective studies | |  | |
| - Primary Outcome: Pregnancy during Covid-19 - Main Conclusion: Cesarean delivery as standard in most of the cases, respiratory insufficiencies of Covid-19 in late pregnancies create a complex scenario. | | | | | | | | | | |
| [94] | | Association of Maternal and neonatal outcomes | Nov 1, 2019 – March 28, 2020 | | systematic review | | 9 case reports | | 92 | |
| - Primary Outcome: Maternal and neonatal outcomes - Main Conclusion: Currently, there are low rates of maternal and neonatal mortality as well as ICU admissions associated with COVID-19. | | | | | | | | | | |
| **COVID-19 and ARDS** | | | | | | | | | | |
| [95] | | Acute Respiratory Distress Syndrome | December 1, 2019, to April 12, 2020 | | Systematic Review | | 4 case reports | | 6 | |
| - Primary Outcome: ARDS in COVID-19-positive patients - Main conclusion: summarized the clinical presentation, laboratory and chest imaging findings, management protocols, and outcome of ARDS in COVID-19-positive patients | | | | | | | | | | |
| **COVID-19 and COPD** | | | | | | | | | | |
| [48] | Prevalence, Severity, and Mortality associated with COPD and Smoking in patients with COVID-19 | | | inception to March 24, 2020 | | Systematic Review  & meta-analysis | | 15 case studies | | 2473 |
| - Primary Outcome: Prevalence of COPD low, but COPD in COVID-19 patients are vulnerable, high risk of severe complications and death - Main Conclusion: COVID-19 infection was associated with substantial severity and mortality rates in COPD | | | | | | | | | | |
| [47] | Impact of COPD & Smoking history | | | Dec 2019 – 22, March 2020 | | Systematic Review  & meta-analysis | | 11 case series | | 2002 |
| - Primary Outcome: Association of active smoking and COPD to COVID-19 - Main Conclusion: COPD and ongoing smoking history is attributable to the worse progression and outcome of COVID‐19 | | | | | | | | | | |
| **COVID-19 and Smoking** | | | | | | | | | | |
| [46] | smoking | | | Until March 17, 2020 | | Systematic Review  & meta-analysis | | 5 case studies | | Ranging from 41 to 1099 |
| - Primary Outcome: Association of smoking and disease outcomes - Main conclusion: smoking is most likely associated with the negative progression and adverse outcomes of the disease | | | | | | | | | | |
| **COVID-19 and Mental Health** | | | | | | | | | | |
| [53] | COVID-19 and mental health | | | Until March 30, 2020 | | Review | | 4 Cross-sectional & Observational studies | | NA |
| - Primary Outcome: Mental health crisis during COVID-19 - Main conclusion: subsyndromal mental health problems are a typical response to the COVID-19 pandemic, need for further research | | | | | | | | | | |
| [96] | Psychiatric and neuropsychiatric effects on COVID-19 patients | | | January 1- April 10, 2020 | | Systematic Review  & meta-analysis | | 79 Case report, cohort studies | | 3559 |
| - Primary outcome: psychiatric signs or symptoms; symptom severity - Main conclusion: possibility of depression, anxiety, fatigue, post-traumatic stress disorder, and rarer neuropsychiatric syndromes in the longer term | | | | | | | | | | |
| [97] | Psychological effects of Covid-19 on healthcare workers | | | up to late March 2020 | | Rapid review & meta-analysis | | 59 Cross-sectional, longitudinal studies | | NA |
| - Primary outcome: successful measures to manage stress and psychological distress - Main Conclusion: Effective interventions of previous outbreaks can help mitigate the psychological distress experienced by healthcare workers by staff caring for patients | | | | | | | | | | |
| **COVID-19 and Diabetes** | | | | | | | | | | |
| [25] | Association with diabetes | | | up to April 2, 2020 | | Review | | 11 case studies | | Varied range |
| - Primary Outcome: Prevalence, pathophysiology, prognosis, and practical considerations - Main conclusion: increased incidence and severity of COVID-19 in patients with diabetes | | | | | | | | | | |
| [27] | Association of diabetes with severity and mortality of COVID-19 | | | April 17, 2020 | | Systematic Review & meta-analysis | | 30 case studies | | 6452 |
| • Primary Outcome: poor composite outcome, including mortality, severe COVID-19, acute respiratory distress syndrome (ARDS), need for intensive care unit (ICU) care, and disease progression.  • Main Conclusion: DM was associated with mortality, severe COVID-19, ARDS, and disease progression in patients with COVID-19. | | | | | | | | | | |
| **COVID-19 and Thyroid** | | | | | | | | | | |
| [42] | Association with thyroid disease | | | June 7, 2020 | | Review | | NA | | NA |
| • Primary Outcome: AITD and Covid-19  • Main Conclusion: no available data suggesting that Association with thyroid disease (AITD) patients are at higher risk of COVID-19, but this requires further research and data analysis | | | | | | | | | | |
| [45] | hyperthyroidism and hypothyroidism during COVID -19 | | | July 2020 | | Review | | NA | | NA |
| • Primary Outcome: Managements of thyroid patients  • Main Conclusion: Detailed guidelines and resources | | | | | | | | | | |
| **COVID-19 and Obesity** | | | | | | | | | | |
| [98] | Obesity with the negative progression of COVID -19 | | | July 2020 | | systematic review | | 3 Retrospective Cohort design | | 806 |
| • Primary Outcome: impact of obesity to the prognosis and disease severity  • Main Conclusion: Obesity is an independent risk leading to the prognosis of disease severity, making obese people vulnerable | | | | | | | | | | |
| **COVID-19 and Cancer** | | | | | | | | | | |
| [99] | Anti-cancer drugs | | | Until May 2, 2020 | | systematic review | | 53 | | NA |
| - Primary Outcome: To understand which anti-COVID-19 drugs could be administered and which not in cancer patients - Main Conclusion: Inconclusive picture on potential preferred treatments for COVID-19 and their interactions with antineoplastic agents, recommendations for the new protocol. | | | | | | | | | | |
| [100] | COVID-19 on cancer patients | | | April 16, 2020 | | systematic review & meta-analysis | | 13 | | 3775 |
| - Primary Outcome: ICU admission and deaths rate in cancer and non-cancer patient groups - Main Conclusion: Presence of cancer in COVID-19 significant impact on developing serious events, i.e., ICU admission, ventilation & mortality | | | | | | | | | | |
| **COVID-19 Vaccines & Treatment** | | | | | | | | | | |
| [83] | Pharmacologic Treatments | | | as of April 2, 2020 | | review | | 291 clinical trials | | NA |
| - Primary outcome: a potential treatment for Covid-19 - Main conclusion: there is no evidence from RCTs that any potential therapy improves outcomes in patients with either suspected or confirmed COVID-19 | | | | | | | | | | |
| [82] | Ongoing clinical trials | | | June 2020 | | review | | 244 | | Varied range |
| - Primary Outcome: Summary of the current trials - Main Conclusion: Summary about the global response in treatment and vaccines | | | | | | | | | | |
| **Covid-19 and Prediction Models** | | | | | | | | | | |
| [90] | Prediction models | | | Living up to May 5, 2020 | | Living  Systematic review | | 107 | | Varied range |
| - Primary Outcome: Prediction models for forecasting future deaths and cases - Main Conclusion: Models have a high risk of bias and should be used cautiously | | | | | | | | | | |
